# Genome-wide identification and expression analysis of *phenylalanine ammonia-lyase* (*PAL*) family in rapeseed (*Brassica napus* L.)

**DOI:** 10.1186/s12870-023-04472-9

**Published:** 2023-10-10

**Authors:** Haiyan Zhang, Xiaohui Zhang, Huixia Zhao, Jin Hu, Zhaoyang Wang, Guangsheng Yang, Xianming Zhou, Heping Wan

**Affiliations:** 1 Sanya Nanfan Research Institute of Hainan University, Hainan Yazhou Bay Seed Laboratory, Sanya, 572025 China; 2 College of Tropical Crops, Hainan University, Haikou, 570288 China; 3 Hubei Engineering Research Center for Protection and Utilization of Special Biological Resources in the Hanjiang River Basin, School of Life Science, Jianghan University, Wuhan, 430056 China; 4 National Key Laboratory of Crop Genetic Improvement, Huazhong Agricultural University, Wuhan, 430070 Hubei China

**Keywords:** Rapeseed (*Brassica napus* L.), *PAL* family, Phylogenetic analysis, Expression profile, Abiotic stress

## Abstract

**Background:**

Phenylalanine ammonia-lyase (PAL), as a key enzyme in the phenylalanine metabolism pathway in plants, plays an important role in the response to environmental stress. However, the *PAL* family responding to abiotic stress has not been fully characterized in rapeseed.

**Results:**

In this study, we conducted a genome-wide study of *PAL* family, and analyzed their gene structure, gene duplication, conserved motifs, *cis*-acting elements and response to stress treatment. A total of 17 *PALs* were identified in the rapeseed genome. Based on phylogenetic analysis, the *BnPALs* were divided into four clades (I, II, IV, and V). The prediction of protein structure domain presented that all *BnPAL* members contained a conservative PAL domain. Promoter sequence analysis showed that the *BnPALs* contain many *cis*-acting elements related to hormone and stress responses, indicating that *BnPALs* are widely involved in various biological regulatory processes. The expression profile showed that the *BnPALs* were significantly induced under different stress treatments (NaCl, Na_2_CO_3_, AlCl_3_, and PEG), suggesting that *BnPAL* family played an important role in response to abiotic stress.

**Conclusions:**

Taken together, our research results comprehensively characterized the *BnPAL* family, and provided a valuable reference for revealing the role of *BnPALs* in the regulation of abiotic stress responses in rapeseed.

**Supplementary Information:**

The online version contains supplementary material available at 10.1186/s12870-023-04472-9.

## Introduction

Phenylpropane metabolic pathway is one of the three important secondary metabolic pathways of plants [1], through which plants directly or indirectly synthesize lignin, flavonoids, coumarins, alkaloids, and other substances containing phenylpropane skeleton [2]. As the key rate-limiting enzyme in the phenylpropane metabolism pathway, Phenylalanine ammonia-lyase (PAL; EC4.3.1.5) catalyzes the deamination of L-phenylalanine to produce *trans*-cinnamic acid [2, 3]. PAL is the first-rate limiting enzyme for phenylpropanoid metabolism, which regulates the anabolism of lignin, flavonoids, isoflavones and alkaloids [4]. In plants, there are many secondary metabolites synthesized through phenylpropane synthesis, such as anthocyanins, lignin, hormones and flavonoids, which play an important role in plant growth, development and adaptation to environmental stress [5]. For example, it was found that *AtPAL1* and *AtPAL2* are highly expressed during nitrogen stress and temperature fluctuations, leading to an accumulation of flavonoids in Arabidopsis [6]. Therefore, PAL plays an important role in plant resistance to environmental stresses.

PAL is widely found in various plants, and has been identified in various plants, such as Arabidopsis [7], *Populus trichocarpa* [8], rice (*Oryza sativa*) [9], walnut (*Juglans regia*) [10], Coleus (*Solenostemon scutellarioides* (L.) Codd) [4] and wheat (*Triticum aestivum* L.) [11]. In most higher plants, there are more than one *PAL* in their genomes. According to previous studies, the number of *PALs* is 4, 7, 9, 4, 12 and 14 in Arabidopsis [6], cucumber [12], rice (*Oryza sativa*) [9, 13], tobacco [14], walnut (*Juglans regia*) [10], and potato (*Solanum tuberosum*) [15], respectively. PAL protein is relatively conserved in plants. Although the quantity of PAL protein varies widely in different plants, their molecular weight is relatively stable, mainly between 275 and 330 kDa [16, 17]. *PAL* family contains multiple *PALs*, and each individual in the family exhibits a distinct expression pattern and a differential response to biotic and abiotic stresses [4].

Rapeseed (*Brassica napus* L.) is the second largest oilseed crop around the world possesses a complex genome. The growth and productivity of rapeseed are significantly influenced by the physiological, biochemical, and molecular levels under various abiotic stress conditions, such as soil salinization [18–20], soil acidification [21] and drought stress [22, 23]. So far, the *PAL* family and key *PALs* responding to abiotic stress have not been fully characterized in rapeseed. Here, we present a genome-wide study to identify the *BnPALs* in the rapeseed genome. To explore the structural diversity and evolution of *BnPALs*, we assessed their phylogenetic relationships, gene structure, conserved motifs and *cis*-acting elements in the promoter region. Additionally, we also demonstrated the expression profile of *BnPALs* treated with NaCl, Na_2_CO_3_, AlCl_3_ and PEG. These findings will not only deepen our understanding of the response of *BnPALs* to abiotic stress, but also facilitate further research on biological functions of the gene family and provide potential gene targets for high-yield breeding under various biotic and abiotic stresses in rapeseed.

## Results

### Identification and characterization of *PALs* in rapeseed

To identify *PALs* in rapeseed, four AtPAL protein sequences were used as queries for BlastP and HMM searches against the rapeseed genome “ZS11”. Consequently, 17 BnPALs possessing the PAL domain were identified (Table 1). Then, these genes are named as *BnPAL1*–*BnPAL17*. The BnPAL proteins above ranged from 210 to 754 amino acids in length (Table 1 and Supplementary file 1). Their relative molecular weights (MW) span from 22.43 kDa to 82.66 kDa. The theoretical isoelectric points (PI) of these proteins lie between 5.01 and 9.21, while their Aliphatic indices range from 73.71 to 97.18. The grand average of hydropathy (GRAVY) for all BnPALs is predicted to be in the range of -0.415 to -0.100, indicating that BnPAL proteins exhibit strong hydrophilicity. Subcellular localization predictions indicate that all BnPAL proteins are cytoplasmic.

**Table 1:** Detail information of *B. napus PAL* gene family

Gene Name	Gene ID	Chromosome	CDS (bp)	Protein length (aa)	Molecular Weight (KDa)	Isoelectric Point	Aliphatic Index	GRAVY	Subcellular Localization
*BnPAL1*	*BnA01g0036810.1*	A01	1491	496	53.25	5.84	88.35	-0.100	Cytoplasm
*BnPAL2*	*BnA02g0046410.1*	A02	1476	491	52.84	5.47	88.04	-0.101	Cytoplasm
*BnPAL3*	*BnA02g0047800.1*	A02	885	294	32.55	6.34	73.71	-0.415	Cytoplasm
*BnPAL4*	*BnA04g0179960.1*	A04	1272	423	47.09	5.76	88.11	-0.339	Cytoplasm
*BnPAL5*	*BnA04g0179970.1*	A04	789	262	27.48	9.21	85.69	-0.126	Cytoplasm
*BnPAL6*	*BnA05g0194150.1*	A05	2265	754	82.66	8.83	85.01	-0.314	Cytoplasm
*BnPAL7*	*BnA05g0218990.1*	A05	2121	706	76.80	5.75	93.10	-0.124	Cytoplasm
*BnPAL8*	*BnA07g0283350.1*	A07	2157	718	78.01	5.70	88.30	-0.182	Cytoplasm
*BnPAL9*	*BnC04g0653260.1*	C04	2175	724	78.60	6.03	90.12	-0.157	Cytoplasm
*BnPAL10*	*BnC04g0661990.1*	C04	1644	547	60.56	5.93	97.18	-0.118	Cytoplasm
*BnPAL11*	*BnC06g0749310.1*	C06	633	210	22.43	5.01	77.62	-0.143	Cytoplasm
*BnPAL12*	*BnC07g0792140.1*	C07	2121	706	76.76	5.78	93.10	-0.130	Cytoplasm
*BnPAL13*	*BnC07g0824100.1*	C07	2124	707	76.79	5.93	89.96	-0.139	Cytoplasm
*BnPAL14*	*BnC07g0824110.1*	C07	2160	719	78.09	5.84	89.82	-0.146	Cytoplasm
*BnPAL15*	*BnC08g0862500.1*	C08	2172	723	78.44	5.97	87.70	-0.184	Cytoplasm
*BnPAL16*	*BnUnng0984250.1*	Scaffold	2121	706	76.76	5.78	93.10	-0.130	Cytoplasm
*BnPAL17*	*BnUnng0984840.1*	Scaffold	2169	722	78.26	6.03	88.35	-0.187	Cytoplasm

**Fig. 1:**
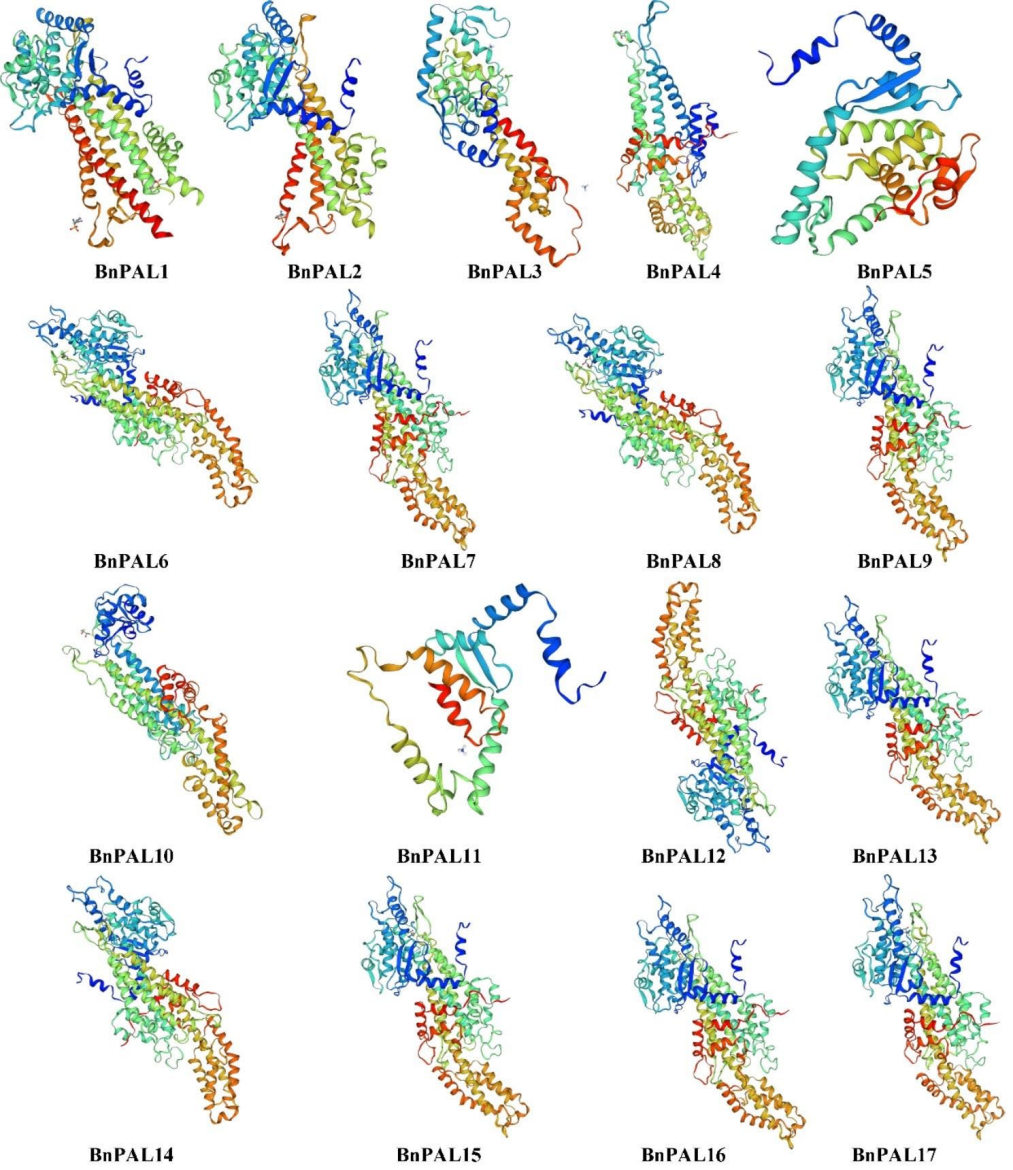
3D structure diagram of BnPAL proteins

Protein secondary structure predictions reveal that all BnPAL proteins comprise four structures: Alpha helix (Hh), Extended strand (Ee), Beta turn (Tt), and Random coil (Cc) (Supplementary file 2). Among the four structures, the proportion of Hh is the highest, ranging from 45.80 to 60.99%, followed by Cc and Ee in order, and the proportion of Tt is the lowest, ranging from 4.02 to 10.31%. Additionally, the 3D protein structures of BnPAL proteins were predicted using SWISS-MODEL. The results highlight that BnPAL6, BnPAL8 and BnPAL14 share high similarity, as do BnPAL7, BnPAL9, BnPAL13, BnPAL15, BnPAL16 and BnPAL17 (Fig. 1).

### Phylogenetic analysis of the *PAL* family

**Fig. 2:**
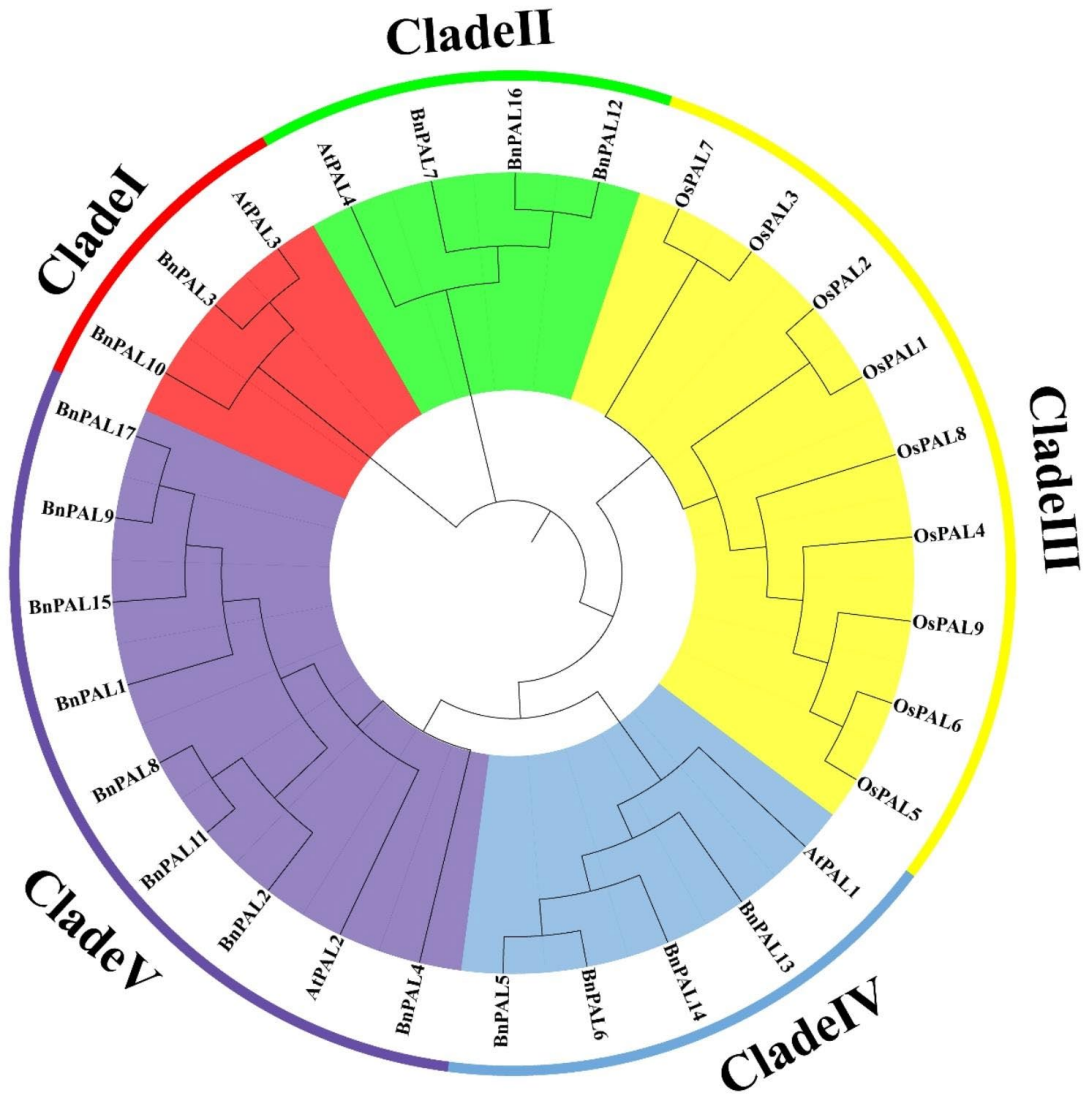
Phylogenetic tree of the *PAL* family in *A. thaliana*, *O. sativa* and *B. napus*. The neighbor-joining tree was generated through the MEGA11 program using the amino acid sequences of the PAL proteins by the neighbor-joining (NJ) method, with 1000 bootstrap replicates. The five major phylogenetic clades are labelled by different colored backgrounds

To elucidate the evolutionary relationships among BnPALs and other PAL proteins, we constructed a phylogenetic tree using multiple sequence alignments of PAL proteins from rapeseed (*Brassica napus* L.), Arabidopsis, and rice (*Oryza sativa* L.) (Fig. 2). The results showed that the PAL proteins were classified into five clades (I, II, III, IV, and V). Phylogenetic analysis results showed that 3, 4, 9, 5, and 9 PAL proteins were clustered into Clade I, Clade II, Clade III, Clade IV, and Clade V, respectively. Notably, all clades incorporated PAL proteins from both rapeseed and Arabidopsis, with the exception of Clade III, which exclusive featured PAL proteins from rice. This distribution underscores the close genetic relationship between PAL proteins from *B. napus* and Arabidopsis.

To further delve into the phylogenetic relationships of PALs within *Brassica* species, we constructed another phylogenetic tree based on PAL proteins from *Brassica napus*, *Brassica rapa*, and *Brassica oleracea*. This analysis revealed that these PAL proteins were grouped into four distinct clades (Fig. S1), aligning with prior classifications [10, 11]. Each of these clades encompassed PAL proteins from all three species, suggesting that BnPALs share a conserved evolutionary trajectory with PAL proteins from both *Brassica rapa* and *Brassica oleracea*.

### Chromosomal locations and synteny evaluation of *PALs*

**Fig. 3:**
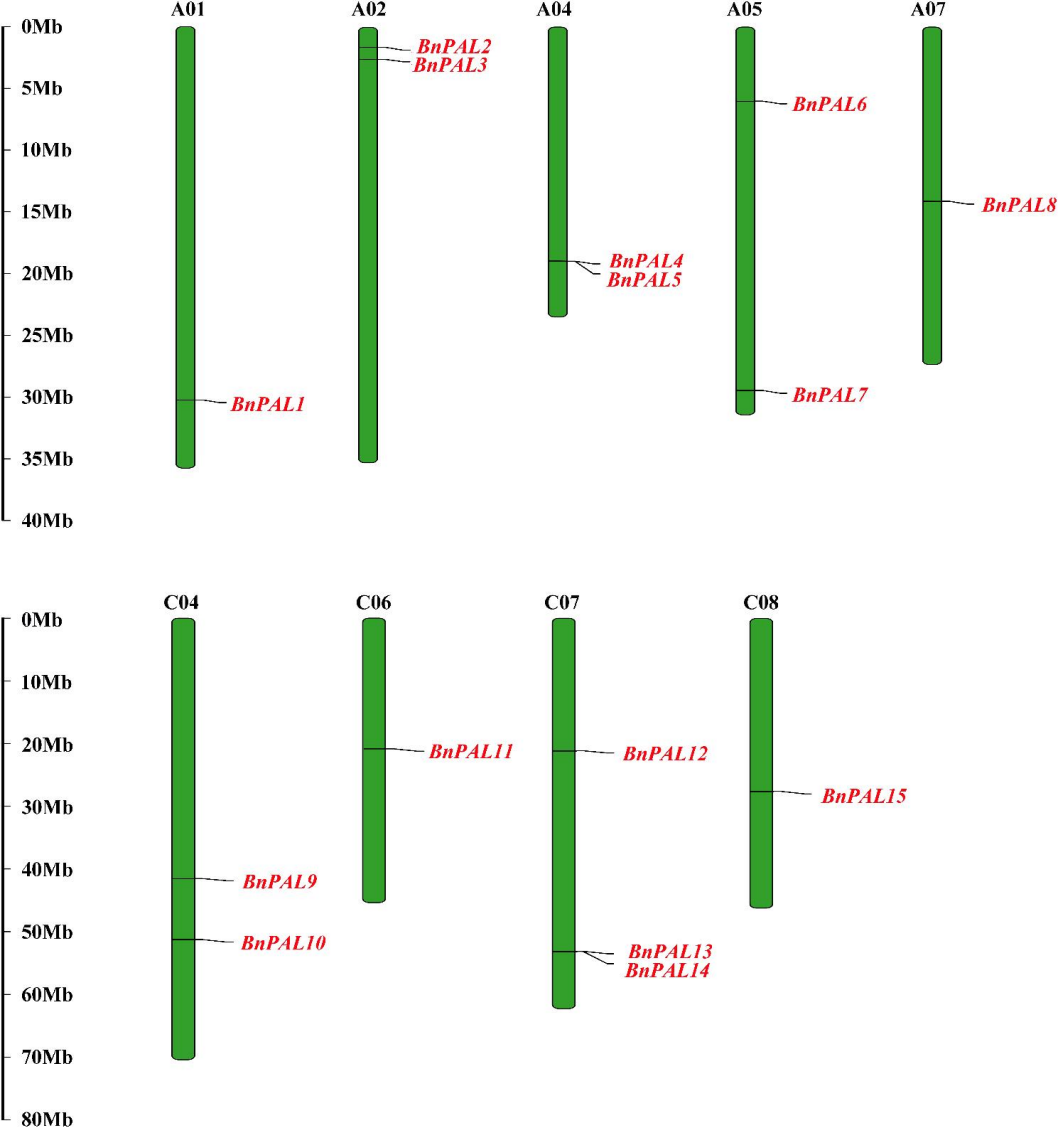
Distribution of *BnPALs* on chromosomes of *B. napus.*. The name of each chromosome is presented at the top of the corresponding green bar, and the gene names are given on the right side of them. The rules on the left indicate the physical position in megabases (Mb)

Chromosomal mapping showed that all the *BnPALs* are unevenly distributed across 9 identified chromosomes (Fig. 3). Notably, *BnPAL16* and *BnPAL17* were situated on unidentified chromosomes. *BnPALs* were mainly located on chromosomes A02, A04, A05, C04 and C07, which contained 2, 2, 2, 2 and 3 genes, respectively. There was only one *BnPAL* on A01, A07, C06 and C08 chromosomes, respectively, while no *BnPAL* was found on the rest chromosomes.

**Fig. 4:**
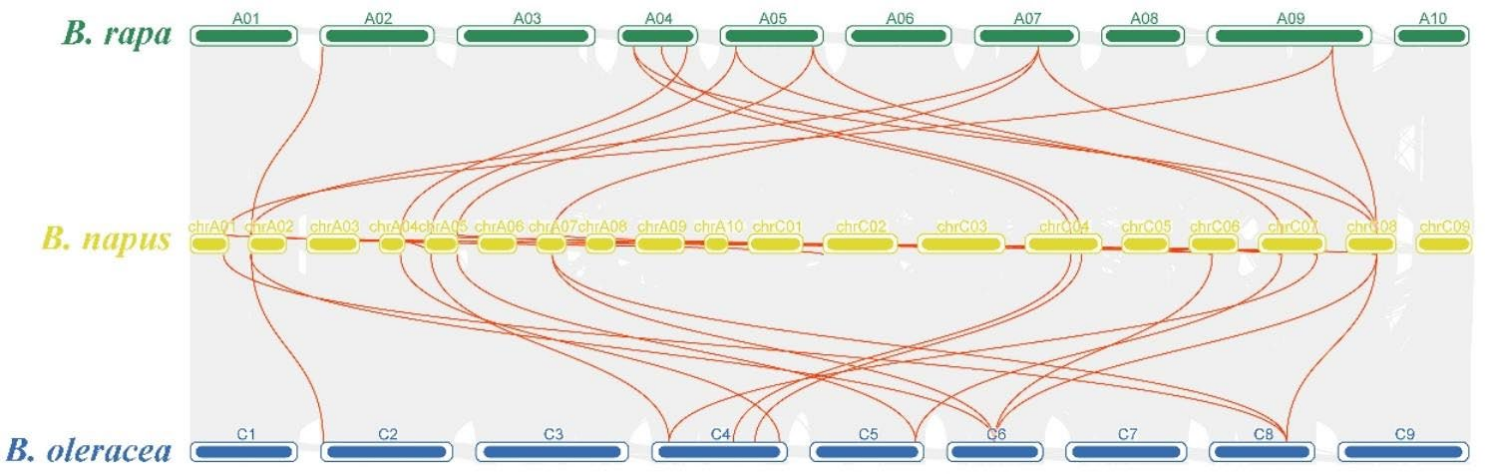
Synteny analysis of *PALs* among *B. napus*, *B. rapa* and *B. oleracea.*. The species names with the prefixes “*B. napus*”, “*B. rapa*” and “*B. oleracea*” indicate *Brassica napus*, *Brassica rapa* and *Brassica oleracea*, respectively. Grey lines in the background are the duplication events among *B. napus*, *B. rapa* and *B. oleracea* genomes, while the red lines indicate the syntenic *PAL* gene pairs. Green, yellow and blue bars represent the chromosomes of *B. rapa*, *B. napus* and *B. oleracea*, respectively. The chromosome number is labelled at the top of each chromosome

To reveal homologous gene functions and phylogenetic relationships between species, a collinearity analysis between *PALs* in *B. napus, B. rapa* and *B. oleracea* was performed. The results showed that the *PALs* of *B. napus* had 14 and 15 homologous gene pairs with the *PALs* of *B. rapa* and *B. oleracea*, respectively (Fig. 4 and Supplementary file 3). Among these homologous gene pairs, *BnPAL15* had a collinear relationship with three *PALs* of *B. rapa* (*BraA04g006280.3 C*, *BraA07g02160.3* C, and *BraA09g044270.3 C*) and two *PALs* of *B. oleracea* (*BolC6t37294H* and *BolC8t50456H*). Some *BrPALs* (*BraA04g006280.3 C*, *BraA05g036420.3 C*, *BraA05g008320.3 C*, *BraA07g021160.3 C* and *BraA09g044270.3 C*) and *BoPALs* (*BolC4t22683H*, *BolC5t34439H*, *BolC6t37294H* and *BolC8t50456H*) were found to be associated with two to four homologous gene pairs in *B. napus*, suggesting that *PALs* experience polyploidization in the process of evolution. In addition, we found that *BnPAL8* and two *PALs* of *B. oleracea* showed a good collinear relationship. Meanwhile, no collinear genes of *BnPAL5, BnPAL11* and *BnPAL14* were detected in *B. oleracea* or *B. rapa*.

### Gene duplication of *BnPALs* in *B. napus*

**Fig. 5:**
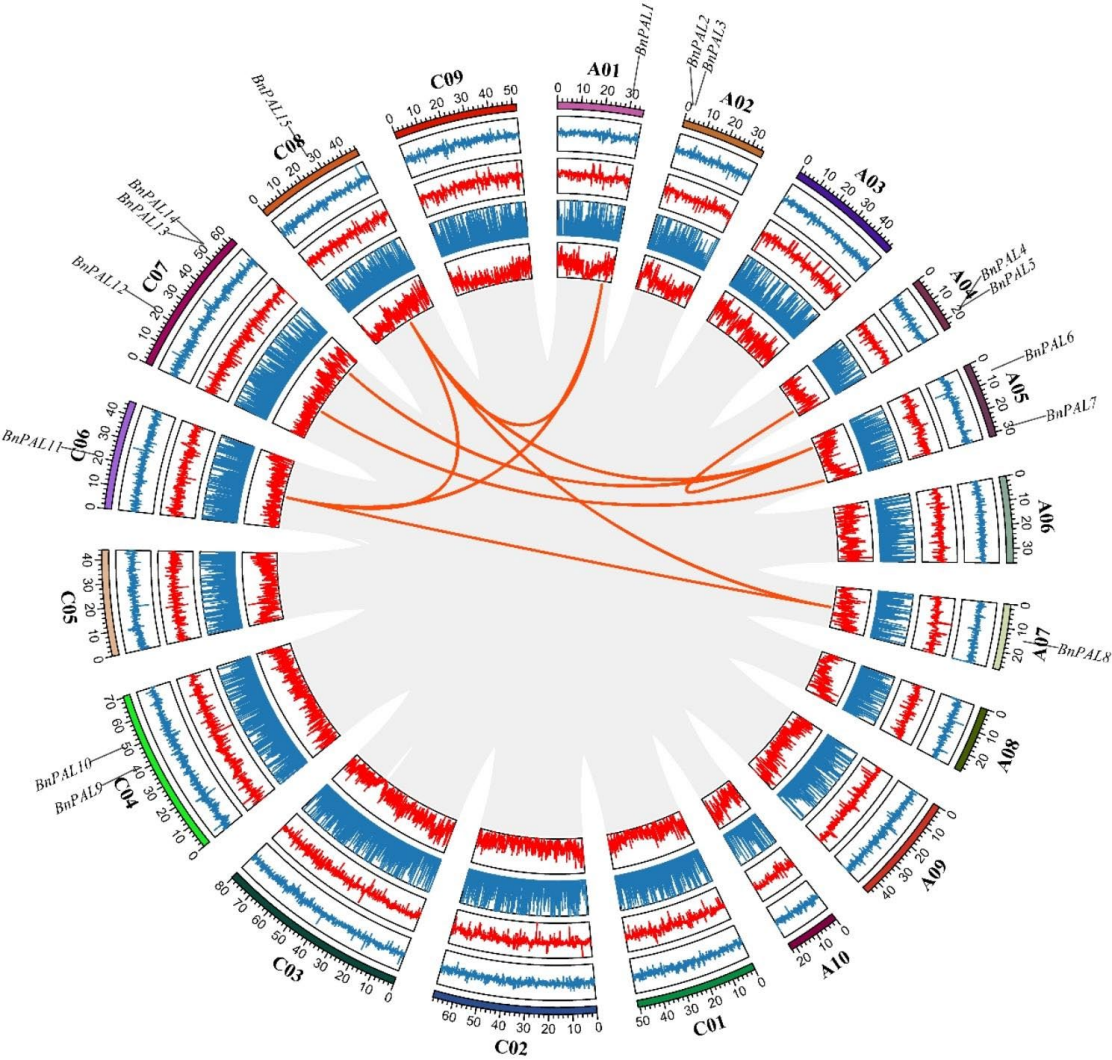
Collinearity analysis between *BnPALs*. The gray lines indicate all synteny blocks between each chromosome and the thick red lines indicate duplicated *PAL* pairs. The circles from inside to outside represent gene density, unknown base ratio, GC ratio, GC skew and the length of chromosome (Mb), respectively. The chromosome name is shown at the bottom of each chromosome. The name and location of *BnPALs* are marked on the respective chromosome

To better understand the evolutionary relationship of *BnPALs* in *B. napus*, gene duplication events were analyzed. The results showed that nine segmental duplication events with seven *BnPALs* in the genome of *B. napus* were identified, which were located on duplicated segments on chromosomes A01, A04, A05, A07, C06, C07, and C08 (Fig. 5). Among them, *BnPAL15* had the most collinearity relationship with other *BnPALs* (*BnPAL1, BnPAL6, BnPAL8*, and *BnPAL11*), while *BnPAL2*, *BnPAL3*, *BnPAL4*, *BnPAL9*, *BnPAL10*, and *BnPAL14* had no collinearity relationship with other *BnPALs* (Fig. 5 and Supplementary file 4).

### Analysis of motifs and conserved domain of BnPAL proteins

**Fig. 6:**
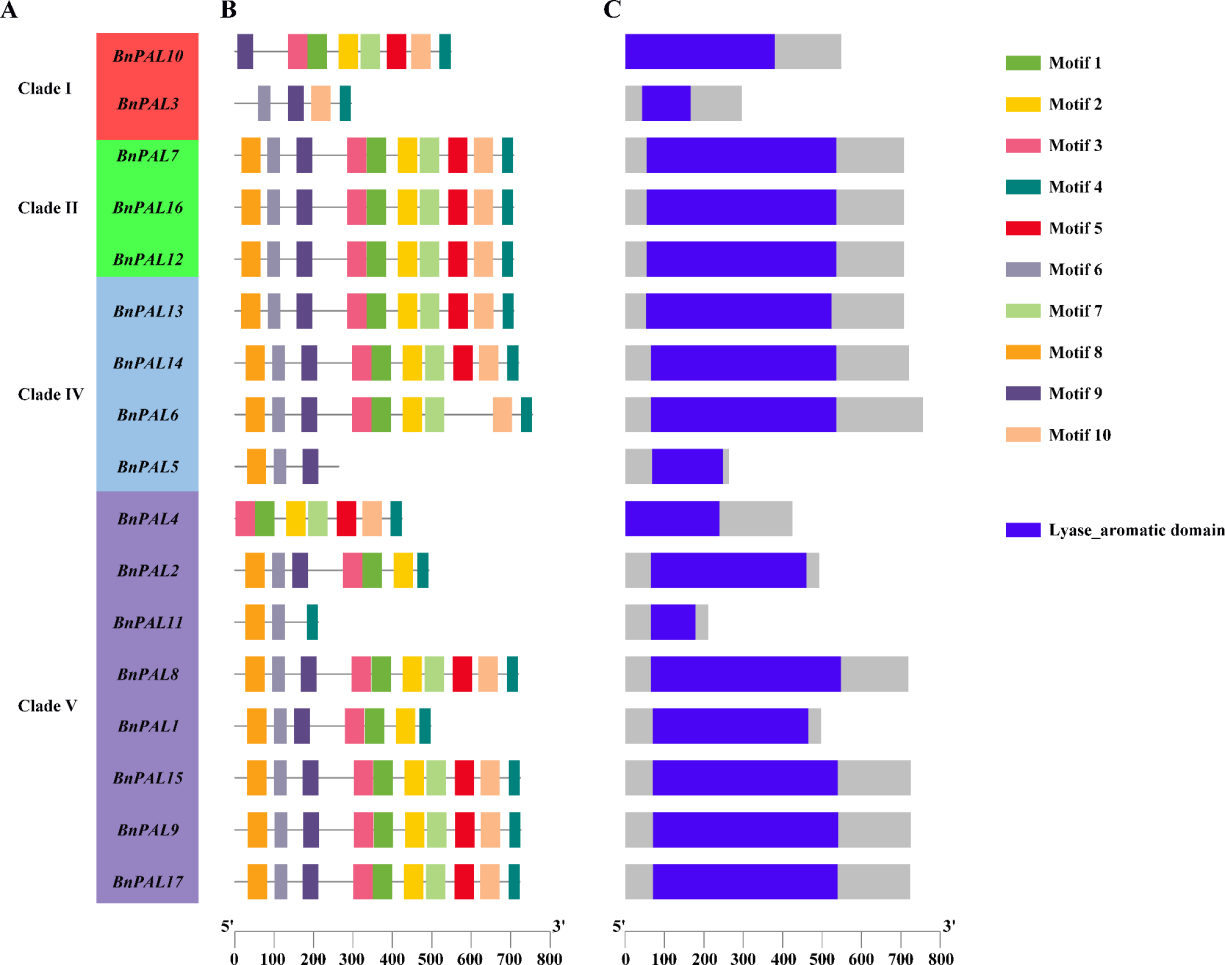
Distribution of motifs and conserved domain in BnPAL proteins. **(A)** Groups of *PALs* in *B. napus*. **(B)** Schematic diagrams of all motifs in BnPAL proteins. Grey lines represent amino acid sequences. Different motifs are annotated by boxes of different colors and numbered 1–10. **(C)** Analysis of conserved domain of BnPAL proteins. The blue box represents conserved domain

To delve into the conservation and evolutionary relationships of PALs in *B. napus*, we analyzed conserved motifs using the MEME program. According to the results, a total of ten conserved motifs, designated as motif 1 through 10, were detected in the *B. napus* PAL protein family (Fig. 6A and B). Most PAL proteins contained motifs 4, 6, 8, and 9, suggesting that the *BnPALs* may encode proteins with similar functions. In clade II, the BnPAL proteins contained all the motifs, and these motifs were arranged in the same order (Fig. 6A and B), indicating that these PAL proteins may have similar biological functions. It is worth noting that different motifs had also been observed in the same clade, indicating that functional differentiation exists in the same clade. In particular, *BnPAL3*, *BnPAL5* and *BnPAL11* contained a considerable small number of motifs, suggesting a potential loss of function or functional differentiation in these genes. To perform the protein conserved domain analysis, the BnPAL protein sequences were subjected to NCBI for cdd-search, the results showed that all BnPAL proteins possess a Lyase _ aromatic domain (Fig. 6C). For most BnPAL proteins, this conserved domain is centrally situated.

### Analysis of *BnPAL* gene structure and *cis* -acting elements in promoters

**Fig. 7:**
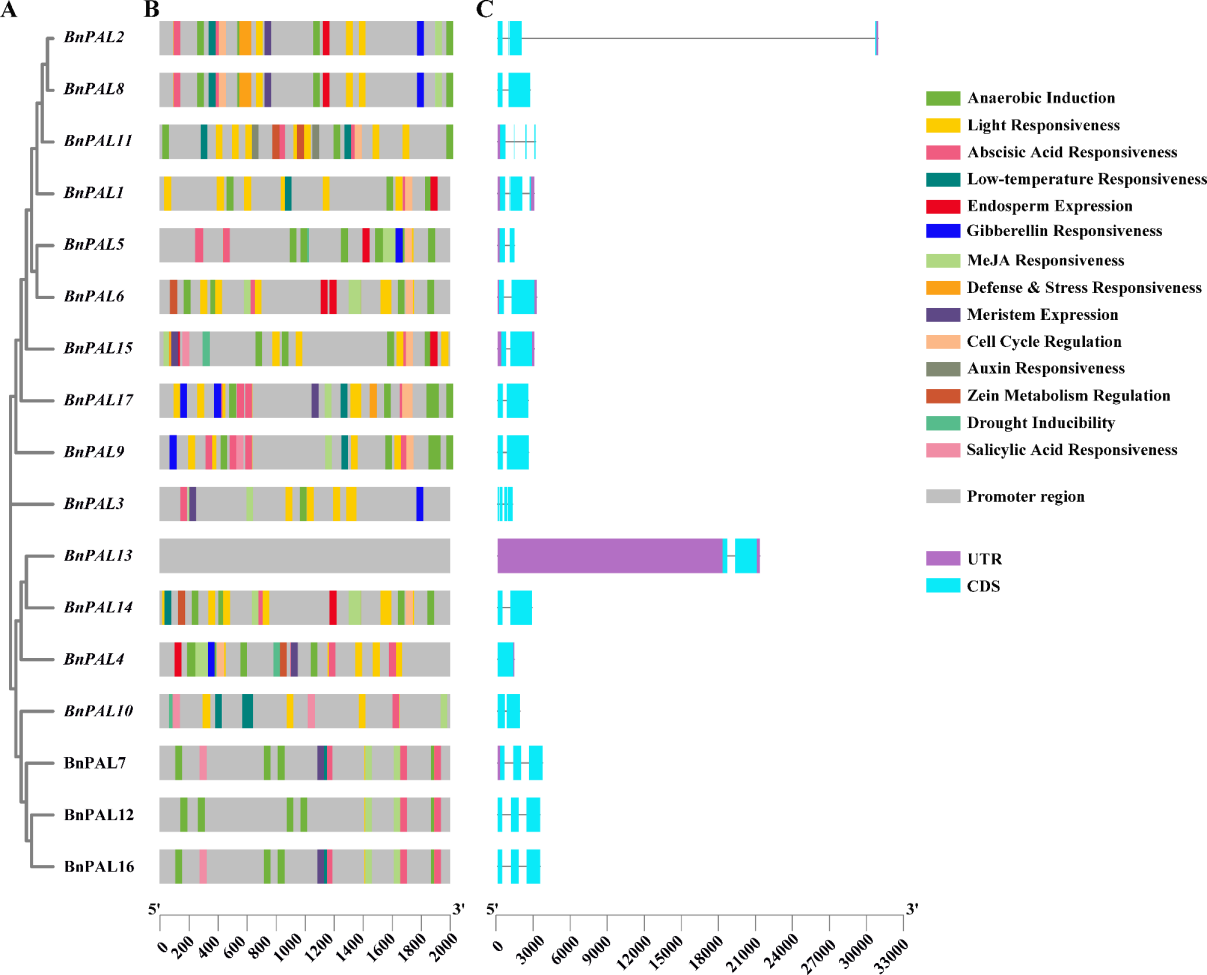
Gene structure and *cis* -acting elements in the promoters of *B. napus PALs*. **(A)** Phylogenetic tree of PALs in *B. napus*. **(B)** Distribution of *cis*-acting elements in the promoter regions of *BnPALs*. Different *cis*-acting elements are annotated by boxes of different colors. **(C)** Gene structure of *BnPALs*. The purple box, blue green box and horizontal line represent UTRs, exons and introns, respectively

To explore the function of *PALs* in plant defense and abiotic stress responses in *B. napus*, a *cis*-acting element analysis was performed in the 2.0 kb promoter region of *BnPALs*. The *cis*-acting elements in the promoter region of *BnPALs* were identified using PlantCARE, and the kind with position of all *cis*-acting elements were marked with different color boxes (Fig. 7A and B). According to the results, the *cis*-acting elements could be mainly divided into 14 categories: abscisic acid responsiveness, anaerobic induction, auxin responsiveness, cell cycle regulation, defense & stress responsiveness, drought inducibility, endosperm expression, gibberellin responsiveness, light responsiveness, low-temperature responsiveness, MeJA responsiveness, meristem expression, salicylic acid responsiveness and zein metabolism regulation. The results showed that all the *BnPALs*, except *BnPAL13*, contained at least one of these *cis*-acting elements. Some *BnPALs* in the same clade had identical distribution of *cis*-acting elements in their promoter region. For example, *BnPAL7* and *BnPAL16* belonging to clade II, as well as *BnPAL8* and *BnPAL17* belonging to clade V, had the same *cis*-acting elements in their promoter regions (Fig. 7A and B). Additionally, we also found that most of the *BnPALs* contained *cis*-acting elements involved in anaerobic induction, abscisic acid, MeJA and defense & stress responsiveness, indicating that *BnPALs* may be induced or suppressed by various biotic and abiotic stresses.

To gain insights into the structural diversity and evolutionary trajectory of *BnPALs*, the gene structure of *BnPALs* was analyzed. According to the results, the number of introns in *BnPALs* varied, ranging from one to four (Fig. 7C). *BnPALs* within the same clade exhibited similar gene structures. For instance, all *BnPALs* in clade II contained three exons and two introns, with the lengths of introns and exons being relatively consistent. Seven *BnPALs* (*BnPAL6*, *BnPAL8*, *BnPAL9*, *BnPAL13*, *BnPAL14*, *BnPAL15* and *BnPAL17*) from clade IV and V displayed analogous gene structure, having the same number of exons and introns. Conversely, *BnPALs* in clade I varied in their number of introns and exons, suggesting diverse gene structures within this group.

### Expression profile of *BnPAL* under abiotic stress conditions

**Fig. 8:**
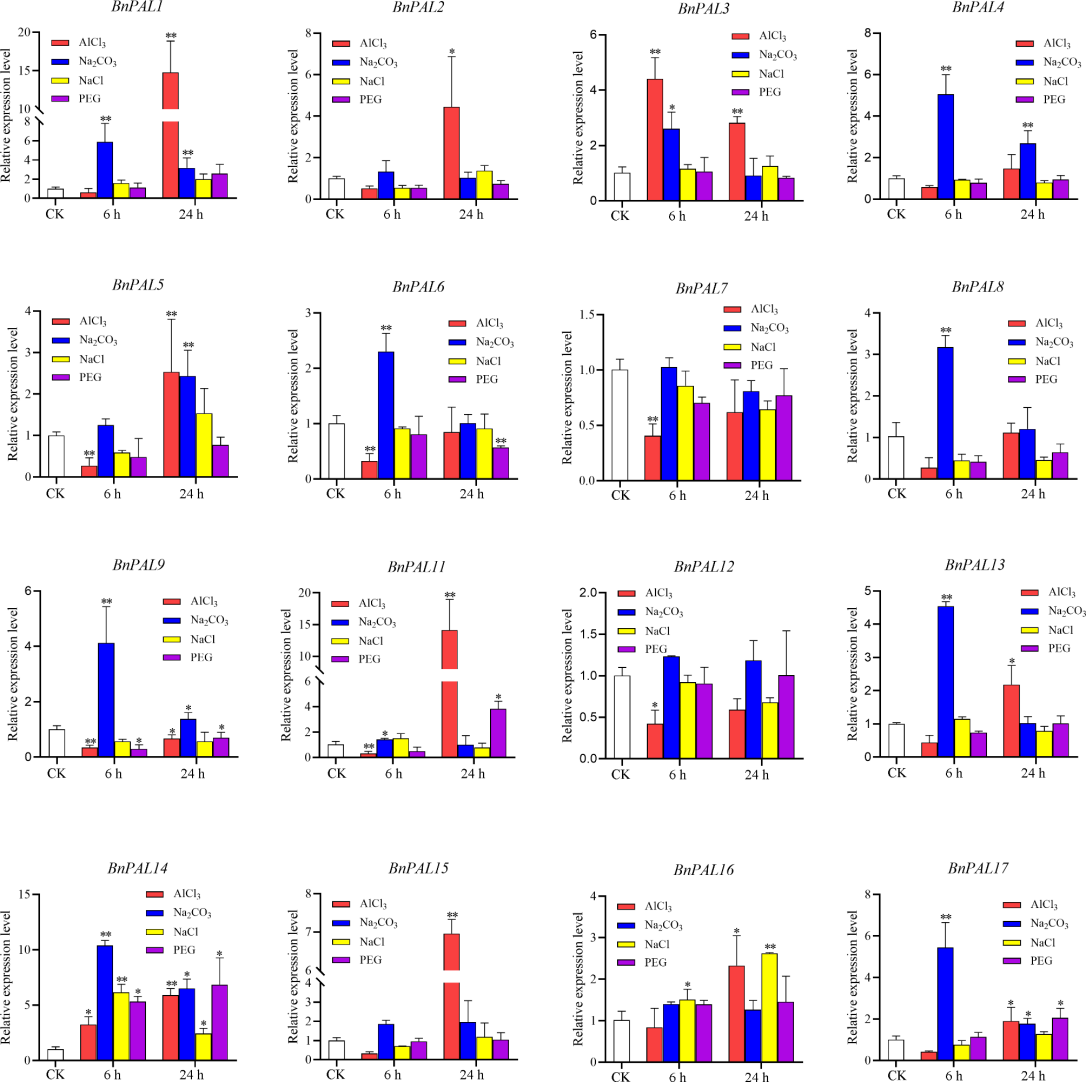
Expression patterns of *BnPALs* under various abiotic stresses. The y-axis represents relative expression, calculated using the 2^−ΔΔCt^ formula. The x-axis represents different stress treatments. Expression profiles of *BnPALs* were obtained under NaCl (1.2%), Na_2_CO_3_ (0.2%), AlCl_3_ (0.5mM) and PEG (PEG6000, 20%) stress conditions, respectively. Samples were collected at 6 and 24 h after stress treatments. Data represent the mean ± standard error for three biological experiments. Student’s *t*-test was used to determine differences. *, *P* < 0.05, **P < 0.01

To further investigate the expression patterns of *BnPALs* under various stress conditions, qRT-PCR was performed to analysis on these genes under NaCl (1.2%), Na_2_CO_3_ (0.2%), AlCl_3_ (0.5mM) and PEG (PEG6000, 20%) stress conditions for 6 and 24 h (Fig. 8). Except for *BnPAL10*, which could not be detected by expression analysis even with five pairs of specific primers, qRT-PCR analysis revealed that the majority of the *BnPALs* were up-regulated (> 2-fold change) under at least one stress condition (Fig. 8). These results suggested that most of the *BnPALs* detected in this study exhibited significant responses to exogenous stressors. Most of the *BnPALs* up-regulated by Na_2_CO_3_ stress reached their peak expression at 6 h after treatment, indicating a rapid response to Na_2_CO_3_ stress. Four genes (*BnPAL1*, *BnPAL11*, *BnPAL14* and *BnPAL15*) were highly up-regulated (≥ 5-fold change) under AlCl_3_ stress, suggesting that they have roles in AlCl_3_ stress response. We also found that the expression level of two (*BnPAL1* and *BnPAL11*) and one (*BnPAL14*) gene under AlCl_3_ and Na_2_CO_3_ stress, respectively, was more than 10 times higher than that of control. Additionally, *BnPAL14* was highly induced in response to all the stress treatments (> 5-fold change) (Fig. 8). According to the results, *BnPAL9* was up-regulated under Na_2_CO_3_ stress but down-regulated under AlCl_3_ and PEG stresses, suggesting that *BnPAL9* might play different roles in response to multiple stresses.

## Discussion

Phenylalanine ammonia lyase (PAL) is an important enzyme involved in the phenylpropane pathway [7, 24]. Recently, there have been numerous reports on the analysis of *PAL* family in various species [9, 10, 13–15, 25–31]. However, the knowledge of *PALs* in rapeseed (*B. napus*), an important oil crop around the world, is still limited. In this study, a genome-wide analysis of *PALs* in *B. napus* was performed, and a total of 17 *BnPALs* were identified. Phylogenetic tree showed that *BnPALs* can be divided into four clades, which was consistent with the results of *PAL* family analysis in wheat [11]. Previous studies have shown that most PAL proteins are located in the cytoplasm [10, 11, 15, 32]. Similarly, the PAL proteins in *B. napus* were also predicted to be located in the cytoplasm (Table 1), suggesting that the subcellular localization of PAL protein in different species is conservative. The secondary structure prediction revealed that BnPAL predominantly consisted of alpha helices and random coils (Supplementary file 2), which was consistent with the results of PAL in other species [4, 33]. Taken together, the *PAL* family is relatively conservative in protein structure and gene evolution.

Compared to *Arabidopsis thaliana*, *Brassica* species including *B. rapa* and *B. oleracea*, have experienced a whole genome triplication (WGT) event during their evolutionary process [34]. *Brassica napus* (*B. napus*), an allotetraploid plant, was derived from natural hybridization between *B. rapa* and *B. oleracea* [35]. Therefore, there is a close genetic relationship between these species. Phylogenetic analysis showed that *PAL*s in Arabidopsis and *B. napus* could be divided into four clades, each of which contained both *AtPAL*s and *BnPAL*s (Fig. 2). Meanwhile, the *PAL*s in rice were divided into a single clade, exhibiting a relatively distant genetic relationship with *PALs* in Arabidopsis and *B. napus*. In addition, phylogenetic analysis among *Brassica* species showed that there is a relatively conservative evolutionary relationship among *PALs* from *B. napus*, *B. rapa* and *B. oleracea*. These results were consistent with the evolutionary relationship among these species.

Since *B. napus* originated from *B. rapa* and *B. oleracea* which have undergone WGT, theoretically, the number of genes in *B. rapa* and *B. oleracea* should be more than twice that of Arabidopsis, and the number of genes in *B. napus* should be more than five times that of Arabidopsis. However, the number of *PALs* in *B. rapa*, *B. oleracea* and *B. napus* is lower than expected (Fig. 4 and Supplementary file 3). These results suggest that part of *PALs* in *B. rapa* and *B. oleracea* were lost after WGT event. Notably, the number of *PALs* in *B. napus* is very close to the sum of *PALs* in *B. rapa* and *B. oleracea*. Additionally, collinearity analysis showed that almost every *BnPAL* can correspond to both *BrPAL* and *BoPAL*, indicating that most *BnPALs* were inherited from their ancestors (*B. rapa* and *B. oleracea*) during hybridization event.

In our study, *BnPALs* are widely dispersed across the genome, similar to *PAL* family studies of most other plants [7, 10, 11, 25]. According to the chromosome mapping results, nearly half of *BnPALs* are located in the A genome, while the other half are located in the C genome (Fig. 3; Table 1). The results of gene duplication analysis showed that most of the duplicated genes corresponding to *BnPALs* in *B. napus* genome A were located in genome C (Fig. 5), indicating that the *BnPAL* family expanded in *B. napus* genome mainly through dispersive duplication.

Recently, many studies have shown that *PALs* are involved in responding to various biotic and abiotic stresses [4, 6, 9, 15]. The *cis*-acting elements distributed in the gene promoter region have been shown to affect plant growth and development, environmental adaptation, and stress resistance [36–39]. Analysis of promoter *cis*-acting elements of the *BnPALs* is conducive to further exploring potential functions. In this study, we analyzed the promoter components of 17 *BnPALs* and identified various *cis*-acting elements. Among these *cis*-acting elements, most genes contained multiple core components that are involved in abscisic acid responsiveness, anaerobic induction, defense and stress responsiveness, drought inducibility and MeJA responsiveness (Fig. 7), indicating that *BnPALs* may play a crucial role in the response to a variety of biotic and abiotic stresses. The promoters of *BnPALs* classified in the same clade seem to contain similar types and numbers of *cis*-acting elements, suggesting that the *BnPALs* in the same clade may have functional similarities. Furthermore, some of the *BnPALs* in the same clade exhibited significant differences in the type and number of promoter components compared to other genes (Fig. 7). Therefore, *BnPALs* in the same clade may also have functional differentiation.

Since PAL plays an important role in plant resistance to environmental stresses, the expression of *PALs* is often induced when plants encounter biotic and abiotic stresses. According to previous studies, *PALs* are induced under cold stress in walnut [40], drought stress in cucumber [12] and lotus [33], high temperature stress in potato [15] and fungus stress in wheat [11]. In our study, the expression level of multiple *BnPALs* increased during various stress stimulations (Fig. 8), indicating that *PAL* gene family in *B. napus* has extensive responses to different stresses. In addition, the expression profiles of nearly all *BnPALs* fluctuated under AlCl_3_ exposure, suggesting a pronounced sensitivity of *BnPALs* to Al^3+^ stress. Intriguingly, while *BnPAL1*, *BnPAL11*, and *BnPAL15* displayed markedly elevated expression compared to the control, *BnPAL9*’s expression dipped significantly below control levels at 24 h under Al^3+^ stress. This hints at potential functional redundancy and differentiation among these homologous genes. The expression level of *BnPAL14* significantly increased under all stress treatments (Fig. 8), implying its multifaceted role in abiotic stress responses. Delving deeper into the biological functions of these genes will undoubtedly shed light on strategies for enhancing yield in rapeseed under various biotic stress conditions.

## Conclusions

In this study, we conducted a comprehensive analysis of the *PAL* family in rapeseed. A total of 17 *BnPALs* were identified and classified into four clades. Further analysis of *BnPALs* including the phylogeny, gene structure, conserved motifs, chromosome localization, gene duplication and *cis*-acting elements provided insight into the molecular evolution of *PAL* family in rapeseed. The expression of most *BnPALs* increased significantly under stress treatment, indicating that the expression of *PAL* family in rapeseed was induced by abiotic stress. These results will contribute to a better understanding of the response of *BnPALs* to abiotic stress and lay the foundation for functional research of *PALs* in rapeseed.

## Methods

### Identification and characterization of ***PALs*** in rapeseed

As described in previous studies, we used BlastP and the Hidden Markov Model (HMM) [11] to identify *PALs* in the *B. napus* Zhongshuang 11 (hereafter referred to as ZS11) genome [41]. The genome database of *B. napus* ZS11 was downloaded from the BnPIR database (http://cbi.hzau.edu.cn/bnapus/index.php). Amino acid sequences of 4 AtPALs (AtPAL1, AtPAL2, AtPAL3 and AtPAL4) were obtained from The Arabidopsis Information Resource (TAIR) database (http://www.arabidopsis.org/). Then the full length of amino acid sequences of these AtPALs were subjected to BlastP against the *B. napus* genome with an e-value of 1 e^− 10^. Meanwhile, the HMM profile of the PAL domain (PF03634) was downloaded from the Pfam protein domain database (http://pfam.xfam.org/). Subsequently, the HMMER 3.1 software (http://www.hmmer.org/) was used to search the *PALs* with default parameters. Finally, combining the above two methods, we identified a total of 17 *PALs* in the rapeseed genome. The genome sequences of *Brassica rapa* (*B. rapa*) and *Brassica oleracea* (*B. oleracea*) were downloaded from BRAD database (https://brassicadb.cn/). Likewise, the *PALs* were also identified in *B. rapa* and *B. oleracea* genomes using the same method.

### Structure, conserved motifs and physio-chemical properties of BnPAL proteins

The physico-chemical properties including molecular weight, theoretical PI, instability index, aliphatic index, and grand average of hydropathy (GRAVY) of BnPAL proteins were evaluated through ExPASy’s ProtParam tool (http://web.expasy.org/protparam/). The subcellular localization of BnPAL proteins was predicted by the WoLF PSORT (https://wolfpsort.hgc.jp/). The protein secondary structure analysis was performed by online tool SOPMA (http://npsa-pbil.ibcp.fr/cgi-bin/npsa_automat.pl?page=npsa_sopma.html). The tertiary structure model of BnPAL protein was developed by SWISS-MODEL website (https://swissmodel.expasy.org/interactive). Gene structures including UTRs, introns and exons were shown by TBtools software (V 1.068; https://github.com/CJ-Chen/TBtools). The conserved motifs of BnPAL protein sequences were identified using the MEME program (https://meme-suite.org/meme/db/motifs) with default parameters [42]. The conserved motif structures were displayed by TBtools.

### Phylogenetic analysis

To explore the evolutionary relationship of the *PALs*, a phylogenetic tree among *B. napus*, *O. sativa*, and *A. thaliana* were constructed. The sequence alignment was executed using Clustal W [43]. The phylogenetic tree was constructed using MEGA 11 software [44] with the neighbor-joining method and 1000 replicate iterations. The Interactive Tree Of Life (iTOL, https://itol.embl.de) was used to visualize the evolutionary tree [45].

### Interspecies synteny analysis and gene duplication

To analyze the genetic relationships of *PALs* in different Cruciferae species, multiple sequence alignments were performed to detect the protein sequences of *B. napus*, *B. rapa* and *B. oleracea* with a similarity of more than 70%. Multiple Collinearity Scan Toolkit (MCScanX; https://github.com/wyp1125/MCScanX) was used to analyze the collinear region with default parameters [46]. Synteny analysis map of *PALs* among *B. napus*, *B. oleracea* and *B. rapa* was illustrated using the python-package JCVI (https://github.com/tanghaibao/jcvi). Gene duplication analysis was performed using the MCScanX program with default parameters, and the location and relationship of duplicated genes were displayed through Circos software [47].

### Analysis of cis-acting elements in the BnPAL promoter

To investigate the putative cis-elements in the *BnPAL* promoter, a sequence of 2000 bp upstream of the initiation codon of the *PAL* in *B. napus* was selected as the regulatory promoter region. Then, these sequences were submitted to PlantCARE website (http://bioinformatics.psb.ugent.be/webtools/plantcare/html/) for cis-acting elements prediction [48], and the results were sorted and displayed by TBtools software [49].

### Plant materials and growth conditions

*Brassica napus* ZS11 seeds were germinated on wet gauze (soaked with water) in a plant growth chamber at 20 to 22 °C and 65% humidity under a long-day condition (16-h-light/ 8-h-dark cycle). The one-week-old seedlings were then transferred into a previously described hydroponic system [19, 50] under the same culture conditions for nearly 20 days until the fourth leaves had extended. For stress treatment research, leaf samples from 4-week-old plants of ZS11 were collected after 6 and 24 h of 1.2% (w/v) NaCl, 0.2% (w/v) Na_2_CO_3_, 0.5 mM AlCl_3_ and 20% (w/v) PEG 6000 treatment. Seedlings without any stress treatment were used as the control. Each treatment includes three biological replications. Leaves were harvested immediately frozen in liquid nitrogen and stored at -80 °C for RNA extraction.

### RNA extraction and quantitative real-time PCR

Total RNA was extracted using the RNA simple Total RNA kit (Tiangen Biotech, Beijing, China) according to the manufacturer’s protocol. cDNA was synthesized with 1 µg RNA from each sample with HiScript® II Q Select RT SuperMix with gDNA wiper (Vazyme, Nanjing, China). Gene-specific primers used for quantitative real-time PCR (qRT-PCR) listed in Supplementary file 5. qRT-PCR was run on the AriaMx real-time PCR system (Agilent Technologies). The following cycling parameters were used: initial denaturation at 95 °C for 5 min; 40 amplification cycles consisting of denaturation at 95 °C for 10s, annealing and extension at 60 °C for 30 s; The melting curve was then tested at 65–95 °C. The internal standard was the *B. napus actin* gene (*BnaA01g27090D*). Three biotic replicates were performed for each sample, and each replicate contained three technical replicates. Relative expression levels were calculated according to the 2^−ΔΔCt^ method [51].

### Electronic supplementary material

Below is the link to the electronic supplementary material.

Supplementary Material 1

Supplementary Material 2

Supplementary Material 3

Supplementary Material 4

Supplementary Material 5

Supplementary Material 6

## Data Availability

The PAL protein sequences of Arabidopsis were collected from the *Arabidopsis* information source (TAIR) database (http://www.arabidopsis.org). The genome sequences of *B. napus PAL* genes were downloaded from the BnPIR database (http://cbi.hzau.edu.cn/bnapus/index.php). All the datasets used and analyzed during the study are include in the article and its additional files.
