# Supplementary figures and images for: Genome-wide identification and expression analysis of phenylalanine ammonia-lyase (PAL) family in rapeseed (Brassica napus L.)
Source: BMC Plant Biol. 2023 Oct 10;23:481. doi: 10.1186/s12870-023-04472-9 (PMC10563225; doi:10.1186/s12870-023-04472-9)

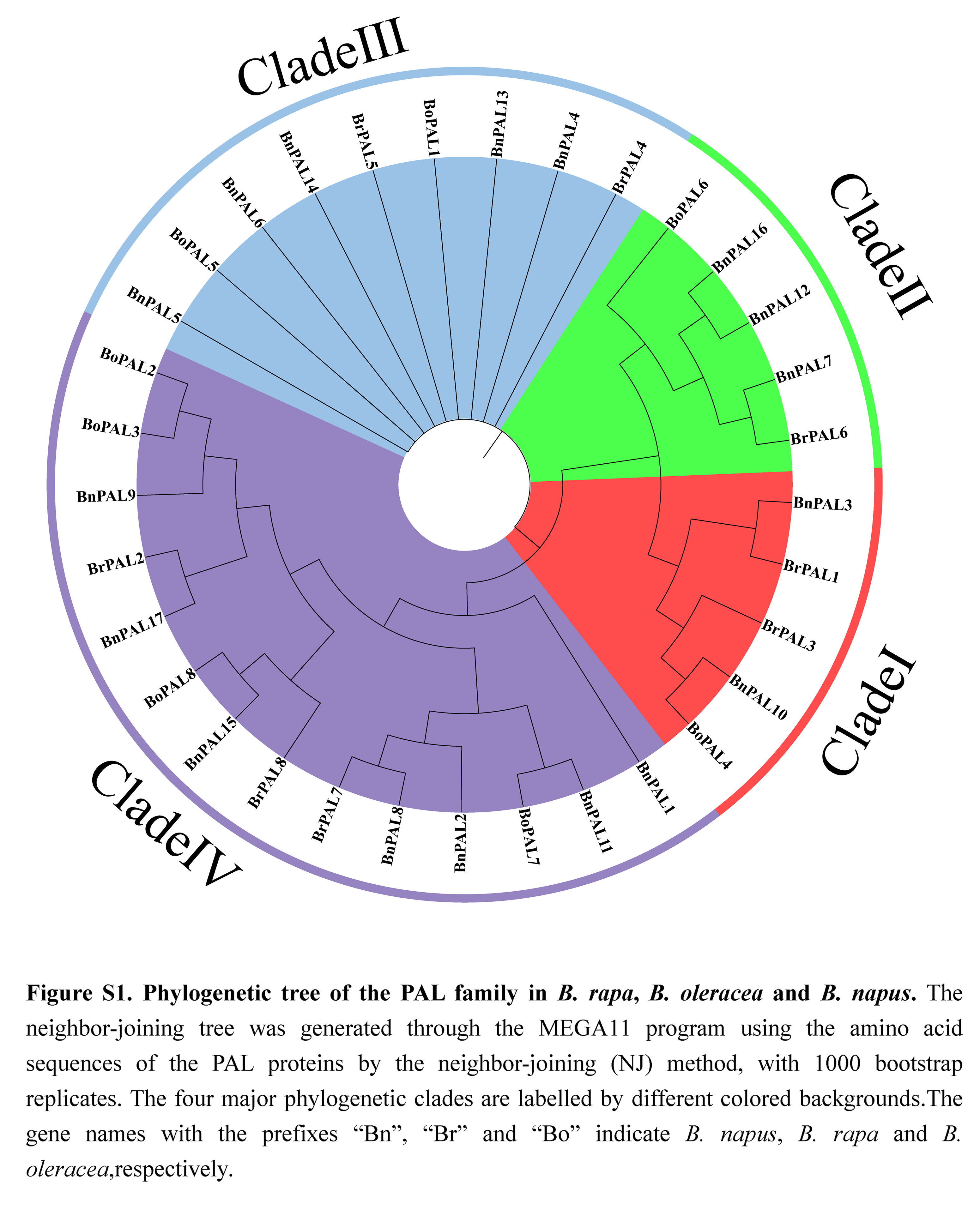

Supplement: Supplementary file 6 — Supplementary Material 6 [file 12870_2023_4472_MOESM6_ESM.tif]
